# Surface roughness on retention of complete dentures using conventional and digital techniques: An in vitro study

**DOI:** 10.6026/973206300212849

**Published:** 2025-08-31

**Authors:** Neeraja B., Kolla Jaswanth, Ruchira Paul, Chamarthy Kundan Chakravarthy, Priyanka Bansal, Adyasha Mohanty

**Affiliations:** 1 Department of Prosthodontics and Crown & Bridge, The Oxford Dental College, Bengaluru, Karnataka, India; 2 Department of Prosthodontics and Oral Implantology, Dr.D.Y.Patil Dental College and Hospital, Pune, India; 3 Kalinga Institute of Dental Sciences, KIIT, Deemed to be university, Bhubaneswar, Odisha, India

**Keywords:** Denture retention, surface roughness, CAD/CAM milling, 3D printing, PMMA

## Abstract

Surface roughness of denture bases plays a crucial role in mucosal sealing and biofilm accumulation, directly impacting denture retention. Therefore, it is of interest to evaluate the effect of surface roughness on retention across three fabrication methods: conventional heat-polymerized PMMA, CAD/CAM milling, and 3D printing. Surface roughness and retention forces were measured and analyzed. CAD/CAM-milled dentures showed the smoothest surfaces and highest retention. A strong inverse correlation between roughness and retention was observed.

## Background:

Retention is a critical factor in the success and functionality of complete dentures, as it ensures stability during mastication and speech. Among the many factors influencing retention, the surface roughness of the tissue-contacting surface of the denture base significantly affects the formation of a mucosal seal and resistance to dislodgement forces [1]. Surface topography also impacts microbial adherence and biofilm formation, which can compromise prosthesis hygiene and patient comfort [2, 3]. Polymethyl methacrylate (PMMA) remains the material of choice for denture fabrication due to its acceptable physical properties, ease of manipulation and cost-effectiveness [4]. Conventional heat-polymerized PMMA dentures often exhibit surface irregularities resulting from processing shrinkage and manual finishing techniques [5]. In contrast, digital fabrication technologies, such as computer-aided design and computer-aided manufacturing (CAD/CAM) milling and 3D printing, have been introduced to overcome these limitations. Milled dentures, fabricated from pre-polymerized PMMA blocks, are reported to possess superior surface smoothness and dimensional accuracy due to minimal polymerization shrinkage [6, 7]. On the other hand, 3D printing offers design flexibility and reduced material waste but may introduce layer-induced surface irregularities depending on the printing resolution and post-processing [8, 9]. The internal surface roughness of complete dentures affects not only retention but also the adaptation of the prosthesis to the mucosa, which in turn influences patient comfort and long-term clinical performance [3].

Surface irregularities can trap air and saliva, disrupting the cohesive and adhesive forces essential for suction-based retention. Furthermore, rough surfaces facilitate microbial colonization, particularly Candida albicans, which is implicated in denture stomatitis and inflammation of the underlying tissues [4]. Hence, smoother internal surfaces are desirable to enhance both mechanical and biological aspects of denture function. CAD/CAM-milled dentures are produced from industrially polymerized PMMA blocks under high pressure and temperature, which results in high polymer conversion and reduced porosity compared to conventional processing [5]. This not only enhances mechanical properties but also significantly reduces surface roughness. Moreover, the digital workflow minimizes human error and ensures consistent quality across dentures. Studies have shown that milled dentures display better adaptation and fit to the tissue-bearing areas, potentially improving retention and stability [6]. In contrast, 3D-printed dentures offer advantages such as rapid fabrication, customization, and cost-efficiency, particularly in large-volume clinical or educational settings. However, the layer-by-layer additive manufacturing process may introduce surface irregularities due to the stair-stepping effect, especially when printed at lower resolutions [7]. Post-processing steps such as polishing and UV curing are often required to improve the surface finish, but these may not fully eliminate surface inconsistencies, which could negatively affect retention and hygiene. Thus, the evaluation of different fabrication techniques in terms of surface roughness and their impact on denture retention remains clinically relevant. Although previous studies have evaluated the mechanical and biological performance of digital dentures, limited research exists on the direct relationship between surface roughness and denture retention across different fabrication techniques [10]. Therefore, it is of interest to assess the influence of surface roughness on the retention of complete dentures fabricated using conventional, CAD/CAM-milled, and 3D-printed PMMA methods.

## Materials and Methods:

This in vitro experimental study was conducted to compare the surface roughness and retention of complete denture bases fabricated using three different methods: conventional heat-polymerized PMMA, CAD/CAM milling, and 3D printing.

A total of ninety standardized maxillary denture bases were fabricated, with thirty specimens assigned to each group:

[1] Group C (Conventional): Denture bases were fabricated using heat-cured PMMA through the conventional compression molding technique. A wax-up was prepared on a standard edentulous maxillary cast, invested, dewaxed, and packed with heat-polymerized acrylic resin. Polymerization was done using a long curing cycle in a water bath.

[2] Group M (Milled): CAD/CAM-milled dentures were designed digitally using 3Shape Dental System software and milled from pre-polymerized PMMA discs using a 5-axis milling machine. Each digital denture base was designed to match the same master model dimensions.

[3] Group P (Printed): Dentures were designed digitally and fabricated using a stereolithography (SLA) 3D printer with light-cured PMMA-based resin. The printing was performed layer-by-layer at 50 µm resolution. Post-processing included isopropyl alcohol rinsing and UV light curing according to the manufacturer's instructions.

All denture bases were finished and polished uniformly using standardized protocols to ensure consistent treatment across groups. Surface roughness (Ra, in micrometers) of the intaglio surface was measured at three points using a contact profilometer (Mitutoyo SJ-210), and the mean value was recorded for each specimen. For retention testing, each denture base was seated onto a silicone-based simulated edentulous maxillary ridge mounted on a custom jig. A vertical dislodging force was applied using a digital force gauge (Lutron FG-5005) connected to a universal testing machine. The maximum force (in Newtons) required to dislodge the denture was recorded as the retention value. All data were statistically analyzed using SPSS version 25. One-way ANOVA was used to compare surface roughness and retention among the three groups, followed by Tukey's post hoc test for pairwise comparisons. Pearson's correlation coefficient was used to assess the relationship between surface roughness and retention. A p-value of <0.05 was considered statistically significant.

## Results:

The study evaluated and compared the surface roughness and retention force of complete denture bases fabricated by three techniques. The mean values for surface roughness and retention are presented in Tables 1 (see PDF) and 2 (see PDF). The highest mean surface roughness was recorded in Group C (conventional PMMA) at 0.27 ± 0.02 µm, followed by Group P (3D-printed PMMA) at 0.19 ± 0.01 µm, while Group M (CAD/CAM-milled PMMA) had the lowest mean surface roughness at 0.12 ± 0.01 µm. The differences among groups were statistically significant (p < 0.001) as shown in Table 1 (see PDF). Retention force followed an inverse trend with surface roughness. Group M showed the highest mean retention force at 32.4 ± 2.3 N, followed by Group P at 25.8 ± 1.8 N, and Group C at 19.5 ± 2.1 N. These differences were statistically significant (p < 0.001), as presented in Table 2 (see PDF). Tukey's post hoc test revealed significant pairwise differences in both surface roughness and retention among all three groups (p < 0.05), as detailed in Table 3 (see PDF). Pearson correlation analysis demonstrated a strong negative correlation between surface roughness and retention force (r = -0.88, p < 0.001), indicating that smoother denture bases tend to have higher retention, as shown in Table 4 (see PDF). These findings confirm that reduction in surface roughness significantly improves the retention of complete dentures (Tables 1-4 - see PDF).

## Discussion:

The current study aimed to evaluate the effect of surface roughness on the retention of complete denture bases fabricated by conventional heat-polymerized PMMA, CAD/CAM milling, and 3D printing. The findings revealed that CAD/CAM-milled dentures exhibited the smoothest internal surface and the highest retention, while conventionally fabricated dentures showed the roughest surface and lowest retention. A strong inverse correlation was observed between surface roughness and denture retention, emphasizing the clinical significance of surface quality in prosthodontic outcomes. Surface roughness critically affects denture retention by altering the quality of the mucosal seal, which relies on intimate adaptation of the denture base to the underlying tissues [1]. Increased roughness may disrupt this adaptation and reduce the effectiveness of adhesion and cohesion forces, thereby compromising denture stability [2, 3]. Additionally, rough surfaces provide niches for microbial colonization, contributing to biofilm formation and predisposing to conditions like denture stomatitis [4, 5]. These biological and mechanical consequences underline the necessity for achieving smoother intaglio surfaces in denture bases [6]. Milled dentures outperformed both 3D-printed and conventional groups in terms of surface smoothness and retention. This is likely attributed to their fabrication from pre-polymerized PMMA blocks under controlled industrial conditions, resulting in higher polymer conversion, minimal porosity, and excellent dimensional accuracy [7, 8]. The subtractive nature of the milling process allows for precise surface finishes, reducing variability and manual errors [9]. On the other hand, heat-polymerized PMMA dentures are prone to irregularities due to polymerization shrinkage, thermal expansion mismatch and finishing inconsistencies [10, 11]. The performance of 3D-printed dentures was intermediate, likely due to the layer-by-layer additive process that introduces stair-step artifacts and micro-voids depending on print resolution [12]. Although advancements in post-processing (e.g., UV curing and polishing) have improved the quality of printed dentures, they may still fall short of the precision and smoothness of milled counterparts [13]. However, the digital workflow of 3D printing offers benefits such as faster fabrication, design customization, and reduced material waste, making it a promising alternative in certain clinical contexts [14]. The inverse relationship between surface roughness and denture retention observed in this study (r = -0.88, p < 0.001) aligns with previous investigations highlighting the significance of surface texture in denture function [15]. These findings support the clinical recommendation to prefer digital fabrication techniques, particularly CAD/CAM milling, to achieve optimal retention and patient satisfaction in complete denture therapy.

## Conclusion:

Denture base surface roughness significantly influences prosthesis retention, with smoother surfaces yielding higher retention values. CAD/CAM-milled dentures demonstrated superior surface finish and retention compared to conventional and 3D-printed methods. Digital fabrication, particularly milling, is recommended to enhance the functional performance of complete dentures.
